# [Retracted] Ultrasound-targeted microbubble destruction-mediated overexpression of Sirtuin 3 inhibits the progression of ovarian cancer

**DOI:** 10.3892/or.2026.9110

**Published:** 2026-04-02

**Authors:** Li Cheng, Dongmei Zhang, Wei Yan

Oncol Rep 46: 220, 2021; DOI: 10.3892/or.2021.8171

Following the publication of this paper, it was drawn to the Editor's attention by a concerned reader that certain of the data showing the results of EdU assay experiments in Fig. 2C on p. 5 were strikingly similar to data that had either already been submitted for publication, or which were already published, in articles in other journals that were written by different authors at different research institutes (a couple of which have subsequently been retracted). Furthermore, there appeared to be one instance of duplication of western blot data comparing Fig. 1D with Fig. 5A.

In view of the fact that the abovementioned data had already been submitted for publication, or were already published, elsewhere before this paper was received at *Oncology Reports*, the Editor has decided that this paper should be retracted from the Journal. The authors were asked for an explanation to account for these concerns, but the Editorial Office did not receive a reply. The Editor apologizes to the readership for any inconvenience caused.

